# Antiviral Activity of Natural Compounds for Food Safety

**DOI:** 10.1007/s12560-024-09605-3

**Published:** 2024-06-17

**Authors:** Irene Falcó, Walter Randazzo, Gloria Sánchez

**Affiliations:** 1 VISAFELab, Department of Preservation and Food Safety Technologies, IATA-CSIC, Valencia, Spain; 2 Department of Microbiology and Ecology, University of Valencia, C/Doctor Moliner, 50, 46100 Burjassot, Valencia Spain; 3 Universidad Internacional de Valencia, Valencia, Spain

**Keywords:** Natural compound, Antiviral activity, Food safety

## Abstract

Gastroenteritis and hepatitis are the most common illnesses resulting from the consumption of food contaminated with human enteric viruses. Several natural compounds have demonstrated antiviral activity against human enteric viruses, such as human norovirus and hepatitis A virus, while little information is available for hepatitis E virus. Many in-vitro studies have evaluated the efficacy of different natural compounds against human enteric viruses or their surrogates. However, only few studies have investigated their antiviral activity in food applications. Among them, green tea extract, grape seed extract and carrageenans have been extensively investigated as antiviral natural compounds to improve food safety. Indeed, these extracts have been studied as sanitizers on food-contact surfaces, in produce washing solutions, as active fractions in antiviral food-packaging materials, and in edible coatings. The most innovative applications of these antiviral natural extracts include the development of coatings to extend the shelf life of berries or their combination with established food technologies for improved processes. This review summarizes existing knowledge in the underexplored field of natural compounds for enhancing the safety of viral-contaminated foods and underscores the research needs to be covered in the near future.

## Introduction

In recent years, human enteric viruses have been recognized worldwide as one of the most significant causative agents of foodborne diseases (Harrison & DiCaprio, 2018; Ruscher et al., 2020). Human enteric viruses are mainly transmitted through the faecal-oral route, as infected people can excrete up to 100 viral particles per gram of feces, facilitating transmission and infection (Arguedas & Fallon, 2004). Among them, human noroviruses are the leading cause of epidemic and sporadic acute gastroenteritis worldwide, making them the most common cause of foodborne illness (Ahmed et al., 2014; Bartsch et al., 2016).

A wide variety of pathogenic viruses can be transmitted through the consumption of contaminated water or food. Among them, human norovirus, sapovirus, astrovirus, rotavirus and adenovirus are responsible for acute gastroenteritis, which manifests abruptly with symptoms such as diarrhea, vomiting, and sometimes is accompanied by fever and abdominal cramps. Severe gastroenteritis caused by human enteric viruses usually requires 2–5 days of treatment focused on maintaining good hydration of the patient (Glass et al., 2023). However, beyond the immediate symptoms and temporary discomfort induced by these enteric viruses, there is an increasing concern over their potential long-term health consequences. Recent studies indicate that some enteric viruses may have long-term impacts on the gastrointestinal system, potentially leading to the development of problems such as irritable bowel syndrome (IBS) and inflammatory bowel disease (IBD) in certain populations (Ansari et al., 2020; Dehghani et al., 2023; Iliev & Cadwell, 2021).

On the other hand, hepatitis A virus (HAV) is the most frequent etiological causative agent of acute hepatitis associated with water and food consumption (Ansari et al., 2020). HAV infection can be completely asymptomatic, as is usually in children under 5 years of age, or it can also cause acute hepatitis, which occurs frequently in adults and has two stages of development: a pre-jaundice stage and a jaundice stage, requiring hospitalization in some cases (Dehghani et al., 2023). Another hepatitis virus transmitted through the faecal-oral route is the hepatitis E virus (HEV), which is zoonotic and considered a re-emerging foodborne pathogen in developed countries.10 According to the World Health Organization (WHO), HEV is estimated to cause 44,000 deaths every year (Iliev & Cadwell, 2021). Depending on the genotypes, the routes of transmission can be either faecal-oral, usually through the consumption of contaminated drinking water or animal meat, or by direct contact with infected animals, typically occurring in developing countries (Pintó Solé et al., 2011). Recently, the presence of HEV has been described in different types of animal meat (such as cow and donkey) and in raw sheep’s milk (Demirci et al., 2019).

The contamination by food handlers or cross-contamination through contaminated surfaces is mainly associated with ready-to-eat products, such as salads, sandwiches, or bakery items, which are prepared or handled raw, or after the foods have been already cooked. Contamination can also occur during pre-harvest. This is the case of shellfish, eventually grown in fecally-impacted waters, as well as for leafy greens and berries contaminated in the fields by pickers or through polluted irrigation waters. The high risk associated with viral infection is due to the fact that all these food items are frequently eaten raw (Kupferschmidt, 2016; Sooryanarain & Meng, 2019). Globally, foodborne hazards cause approximately 600 million illnesses annually, with the human norovirus being responsible for 120 million cases attributed to water and food (Daniels et al., 2009). In 2021, there were 4005 officially notified foodborne outbreaks (FBOs) in the European Union (EU), showing a 29.8% increase compared to 2020. Norovirus, including other caliciviruses, ranked as the third most frequently reported agents causing FBOs, according to reports from 14 EU member states and two non-member states. France had the highest contribution with 112 outbreaks. Hepatitis A caused FBOs in six member states, with one strong-evidence outbreak in Norway and a major weak-evidence outbreak in Czechia. Additionally, Hepatitis E FBOs were reported in Belgium and Switzerland, with a severe outbreak documented in Switzerland (Authority & European Centre for Disease Prevention and Control, 2022).

Enteric viruses present high stability to environmental stressors, providing them with long survival rates under extreme conditions. This resistance is also observed during exposure to inactivation processes that are commonly applied in the food industry (e.g., thermal treatments, chemical disinfection) and along water (re)cycle processes (e.g., wastewater and drinking water treatment plants, WWTPs and DWTPs). Moreover, only a very low infectious dose is needed to cause a viral infection. For example, Rotavirus has an infectious dose of 0.9 focus forming units (ffu) (Ward et al., 1986), while Poliovirus Type 1 and Type 3 have doses of two plaque-forming particles and 1 TCID_50_, respectively (Katz & Plotkin, 1967). Norovirus, which has been extensively studied, has an infectious dose of 18 viruses (Teunis et al., 2008). It is also noted that the infectious dose of HAV remains uncertain, with estimates ranging from one virion according to Grabow (1997) to an assumed range of 10–100 virions based on research by Venter et al. (2007). These features represent the key factors contributing to FBOs by maintaining viral infectivity on surfaces (fomites) and in food products, spreading viral particles, and facilitating cross-contamination, finally resulting in highly transmitted diseases (Kuusi et al., 2002).

Furthermore, the economic impact of foodborne diseases attributed to enteric viruses, including health costs and productivity losses, remains considerable (Ahmed et al., 2014; Bartsch et al., 2016; Havelaar et al., 2015). As such, the development of effective preventive measures and alternatives to conventional food processing technologies is crucial for effectively tackling these pathogens.

## Methodologies Applied to Assess the Antiviral Activity of Natural Compounds for Food Applications

To date, alcohols, quaternary ammonium compounds, and chlorine have been the most commonly used and studied sanitizers in food industry (Falcó et al., 2023a, b; Ogunniyi et al., 2019). However, the EU and the USA are intending to limit their use, especially chlorine-based sanitizers, due to concerns about chemical residues (WHO, 2013). Searching for alternative compounds with lower risk for consumers, natural compounds with antimicrobial and antiviral activity have emerged as promising candidates for use in food processes. Consequently, the evaluation of the antiviral activity of these natural compounds in food matrices has seen significant developments in recent years (McLeod et al., 2022). Traditionally, these assessments involve contaminating a sample with a known amount of virus and measuring the viral titer after exposure to specific conditions and/or compounds. Statistical analyses are then applied to determine the significance of viral decay. However, these approaches rely on viruses that can be cultured in cell lines and quantify through infectivity assays. This limitation restricted the range of viruses and strains that could be studied, such as human norovirus, and HAV and HEV wild type strains, for which in-vitro cultivation systems remain challenging (Estes et al., 2019; Fu et al., 2019; Kanda et al., 2020; Todt et al., 2018). Virus detection through cell culture mainly relies on observing cytopathic effects, followed by quantification using plaque assays, the most probable number, or tissue culture infectious dose (TCID50) using surrogate viruses or cell-culture adapted strains. In recent years, significant progress has been made in developing systems capable of cultivating human noroviruses in-vitro using three-dimensional enteroids generated from human intestinal stem cells (known as human intestinal enteroids, HIE) (Ettayebi et al., 2016). Additional alternative models to cultivate human norovirus have been recently described, including the zebra fish model (Dycke et al., 2019; Tan et al., 2023) and human salivary glands (Ghosh et al., 2022). However, it is important to acknowledge that some limitations still exist, which must be addressed before these models can be used on a routine basis (Costantini et al., 2018; Ettayebi et al., 2016). To overcome these limitations, researchers turned to cultivable surrogates such as feline calicivirus (FCV), murine norovirus (MNV), and Tulane virus (TV) to assess human norovirus survival following inactivation processes. However, the suitability of these surrogate models has been questioned and requires further confirmation (Bae & Schwab, 2008; NACMCF Executive Secretariat, 2016).

Alternative methods for assessing the binding ability, integrity of the capsid, or the integrity of the nucleic acid have been proposed as indirect measurements of viral infectivity. Saliva and porcine gastric mucin (PGM) contain multiple human histoblood group antigens (HBGA) recognized as (co-)receptors for human norovirus (Tian et al., 2005). Thus, saliva and PGM have been used to selectively recover potentially infectious human noroviruses (Dancho et al., 2012; DiCaprio, 2017). These binding assays have been used to evaluate the antiviral activity of grape seed extract (GSE) and green tea extract (GTE) against human norovirus and virus-like particles (VLPs) of human norovirus (Falcó et al., 2019a, b, c; Li et al., 2012). Conversely, viability markers, such as photoactivatable dyes (e.g., propidium and ethidium monoazide) or metal compounds like platinum chloride, can penetrate damaged or altered viral capsids and intercalate the nucleic acid, thereby interfering with PCR amplification. This allows for the estimation of potentially infectious viruses. Thus far, viability RT-qPCR has been successful in detecting the inactivation of human norovirus genogroup I (GI) and GII exposed to epigallocatechin gallate, a derived compound from GTE (Falcó et al., 2017). However, a recent study reports significant limitations of viability RT-qPCR compared to replication in HIE for inferring human norovirus inactivation (Wales et al., 2024). An additional analytical tool is the full-length or long-range RT-PCR which has been used to estimate genomic integrity as a proxy for viral infectivity (Pecson et al., 2011; Raymond et al., 2023). However, as amplification efficiency decreases with fragment size, its robustness and sensitivity have not always been confirmed.

### Evaluation Methods for Antiviral Activity in Formulations

The absence of an official regulation specifically designed to evaluate the antiviral activity of natural compounds for food applications forced scientists to use or adapt home-made protocols. Increasing numbers of standards are being released for viruses; however, many fields of application remain uncovered. The majority of standards in applied virology are derived from those originally developed for bacteria. For example, the ISO 12353, developed to determine the bactericidal activity, served as framework for ISO 14476, which establishes the methods for virucidal activity (ISO, 2015). However, due to the inherent differences among microorganisms, many bacterial standards are not suitable for viruses.

As a preliminary procedure to assess the antiviral activity of natural compounds, the evaluation involves a solubilization/emulsification step of the natural compounds at a given concentration. This is followed by a viral inoculation into the solution, a waiting/contact time during which the active compound in the solution exerts its activity, and finally, a neutralization step using a solution (referred to as a neutralizer) to stop the compound’s action. In the food industry, a relevant variable to considered is the organic load of the solution in which the natural compound is expected to be used. To mimic this, fetal bovine or calf serum is added to the solution at a typical concentration of 10% v/v to mimic the organic load of dirty surfaces. A comprehensive investigation should also monitor additional factors including temperature, solubility of compounds, static or agitation conditions, and pH. After neutralization, viral inactivation should be determined by directly titrating the solution in cell culture. The experimental design requires the inclusion of proper controls: a positive-control solution with the virus only (without the active compound) must be tested under the same experimental conditions to rule out potential viral decay, and a negative-control solution (without the virus and without the natural compound) must be tested to check any effect on the cells. In addition, assessment of cytotoxicity can be performed by monitoring changes in cell appearance, such as cell enlargement, granularity, rounding and plaque detachment over time, while the MTT assay provides a rapid and sensitive method for evaluating antiviral agents. Inclusion of a neutralization control is also necessary ensures cessation of antiviral activity at a specific time point.

Considering all these variables, the resulting protocols differ in the solubilization, exposure, and titration techniques adopted depending on the specific antiviral compound being tested and the intended food application.

### Methods for Assessing the Antiviral Activity of Antiviral Materials

The broad range of applications and diverse nature of materials made the establishment of standard methods for evaluating their antiviral properties challenging. Consequently, researchers have adapted existing methods designed for evaluating the antibacterial activity of antibacterial- and antifungal-treated plastics, as well as other non-porous surfaces (e.g., ISO 22196, and JIS Z 2801 standards), to test the antiviral properties of materials (Pecson et al., 2011; Wales et al., 2024). In 2018, the ISO 16777 and ISO 21702 standards were specifically release to assess the virucidal capacity of materials. While these standards are not specifically intended for food industry applications, they both align with the goal of testing the antiviral effectiveness of materials incorporating natural compounds against foodborne viruses (ISO, 2015; Raymond et al., 2023).

The assessment of the antiviral properties of polymeric materials typically involves viral inoculation onto the material, a waiting/contact period for the active compound in the polymeric material to exert its antiviral effect, and a subsequent recovery step using a neutralizing solution or swabbing. Control materials without active compounds are included for comparison. However, challenges arise when applying this approach to coatings due to the gel-like nature of the biopolymers commonly used. To address this, ISO 14476 has been successfully adapted to test the antiviral activity of polymer gels (Falcó et al., 2019a, b, c).

In addition to the variations mentioned above, real-world applications may introduce additional variables, as food matrices can interact with antiviral compounds, requiring higher compound concentrations for the same effect reported in-vitro. Consequently, while laboratory tests may report high antiviral activity, the translation of these findings into reduced foodborne viral transmission risk in practical scenarios remains an underexplored area pending to be comprehensively assessed in the future. For instance, antiviral activity observed in laboratory tests involving silver nanoparticles on coupons did not consistently replicate when applied to highly turbid surface waters, possibly due to interactions with nonspecific particles (Luceri et al., 2023). This highlights the evolving nature of antiviral material assessment in addressing scientific challenges and real-world complexities.

## Natural Compounds

The search for novel alternatives to traditional chemical and physical processing technologies for food conservation and decontamination is one of the main objectives of the WHO and the food industry (Kuusi et al., 2002). In addition to the increasing consumer demand for worthwhile and “green” alternatives to chemicals, a special interest has emerged in the use of natural compounds. Generally, natural compounds display low toxicity and a lack of secondary effects, as most of them are Generally Recognized as Safe (GRAS) substances (Havelaar et al., 2015). The moderate production costs and their abundance in raw materials and by-products make natural compounds an important source of antimicrobials and a great alternative to chemicals, allowing them to be used as harmless formulations for preserving food safety (Falcó et al., 2019a, b, c; ISO, 2019).

Several studies have investigated the antiviral activity of natural products without characterizing their chemical composition, being the compounds responsible for viral inactivation unknown (Luceri et al., 2023). For instance, date syrup and propolis blocked norovirus VLP binding to HBGAs, caused by the aggregation of viral particles as indicated by dynamic light scattering (Ayaz et al., 2019).

For decades, several secondary metabolites present in plant extracts, otherwise known as phytochemicals, have been extensively studied due to their antimicrobial properties. Furthermore, their synergetic activity with many drugs to combat multi drug-resistant pathogens has been reported (Burt, 2004; El-Saber Batiha et al., 2021). For these reasons, studies on natural compounds propose them as an alternative method to control enteric virus contamination. Among plant extracts, the *Ephedra herba* crude extract was demonstrated to inhibit human norovirus infection in post-entry steps using the HIE model (Hayashi et al., 2023). Silvestrol, a secondary metabolite from *Aglaia foveolata* plant, is known for its specific inhibition of the RNA helicase and recently demonstrated to block HEV replication in a dose-dependent manner at low nanomolar concentrations acting additively to ribavirin (Kanda et al., 2020; Luceri et al., 2023). Also, extracts approved by US Food and Drug Administration as food additives in beverages demonstrated antiviral activity against norovirus surrogates, such as the case of *Quillaja saponaria* Molina (Ruoff et al., 2022).

Phytochemicals, can be divided into different categories: organic acids, essential oils (EOs), polypeptides, polyphenols, proanthocyanins, saponins, polysaccharides and sulfur compounds. In the last decade, antiviral studies have focused primarily on polyphenols and Eos (Ayaz et al., 2019; Battistini et al., 2019; Bozkurt et al., 2014; Joshi et al., 2023). For most of them, the specific mechanisms behind the antiviral effect are not fully understood, but the damage of different structures involved in infection (viral capsid or host cell membranes), which subsequently affects viral attachment to host cells, has been frequently observed (Knight et al., 2013; Zhang et al., 2012a, b). As summarized in Table 1, numerous natural compounds have been evaluated against enteric virus or surrogates.

**Table 1:** Major groups of natural compounds categories evaluated against human enteric viruses or their surrogates

Category	Main natural compound	Viruses evaluated	Method	Reductions (log)	References
Polyphenols	Grape Seed Extract	FCV	PFU	4.61	Amankwaah (2013)
		MNV		1.73	
		HAV		3.20	
	Green tea extract	Human Norovirus	RT-qPCR	0.31	Falcó et al. (2019a, b, c, 2020), Randazzo et al. (2020)
		MNV	TCID_50_	UDL	
		HAV			
	Immature persimmon fruit	MNV			Su et al. (2010a, b), Zhong et al. (2012)
		HAV		NDT	
	*Posidonia Oceanica*	FCV		3.4	Méndez et al. (2021)
		MNV		2.6	
	*Ziziphora hispanica*	FCV		4.21	Benito-González et al. (2019), Duque-Soto et al. (2022)
		MNV		NDT	
	*Thymus longiflorus*	FCV		2.25	
		MNV		NDT	
	*Origanum bastetanum*	FCV		2.21	
		MNV		2.16	
	*Luma apiculata* (DC.) Burret			UDL	Duque-Soto et al. (2022)
Essential oils and compounds thereof	Carvacrol	FCV			Moussaoui (2013)
		MNV			
		HAV		1.0	
	Zataria	FCV		NTC	Battistini et al. (2019)
		MNV		0.1	
		HAV		0.4	
	Lemongrass			2.8	Gilling et al. (2014)
	Mint	MNV		0.9	
	Oregano	FCV		0.3	Zhang et al. (2012a, b)
		HAV		0.1	
	Thymol	MNV		0.5	Kovač et al. (2012)
		HAV		NDT	
	Orange			2.1	Kim et al. (2017), Kovač et al. (2012)
	Grapefruit			2.9	
	Rosemary			3.0	
	Clove	FCV		3.8	Zhang et al., (2012a, b)
		MNV		0.8	
	Allspice	FCV			Elizaquível et al. (2013)
		MNV			
Polysaccharides	*Stevia rebaudiana*	RV	Binding	28%*	Takahashi et al. (2001)
	Chitosan	FCV	PFU	> 3.1	Amankwaah (2013)
	β-glucans	MNV	TCID_50_	> 3.30	Pérez-Bassart et al. (2024)
	Carrageenans			3.2	Falcó et al. (2019a, b, c)
		HAV		2.7	
Organic acids	Citrate	Human Norovirus	Binding	3.60**	Girond et al. (1991), Koromyslova et al. (2015)
	Betulinic acid	FCV	TCID_50_/mL	4.17	Li et al. (2013)
Proteins	Lactoferrin	MNV RV	PFU	UDL	Ishikawa et al. (2013), Kvistgaard et al. (2004)
				0.6	

*FCV* feline calicivirus; *MNV* murine norovirus; *HAV* hepatitis A virus; *RT* rotavirus; *UDL* under detection limits; *NTD* no titer decrease

*% RV Binding, **OD450

### Natural Compound Categories

#### Polyphenols

This group of phytochemicals is one of the most important due to its antioxidant, anticarcinogenic or neuroprotective properties, among others. Consequently, the trade of polyphenols as functional food has increased in the last decade (Cozzi et al., 2023; Zhang et al., 2012a, b). Foods such as cranberries, grapes, or pomegranates are rich in polyphenols. Different studies have reported that cranberry and pomegranate juices, GSE, and GTE have strong antiviral activity against human norovirus and its surrogates, includingFCV, MNV, and TV, as well as HAV (Amankwaah, 2013; Chiang et al., 2003; Falcó et al., 2020; Li et al., 2012; Randazzo et al., 2020; Yilmaz & Toledo, 2004). GSE is recognized for containing a minimum of 95% flavanols among polyphenols, with 12% being highly active monomeric proanthocyanidins and 82% oligomeric proanthocyanidins. GTE primarily comprises catechins, a class of flavonoids known for their antimicrobial activity. Epigallocatechin-3-gallate (EGCG) and epicatechin gallate (ECG) have been identified as the most effective antiviral compounds among those contained in GTE (Amankwaah, 2013; Randazzo et al., 2020). Although the specific antiviral components contributing to the antiviral activity have not always been identified, it seems that different compounds included in the natural extracts play a synergistic role in exerting the antiviral effect. For instance, the antiviral mechanism of action for cranberry polyphenols, as described by Lipson and collaborators, relies on the prevention of virus replication inside the host cells (Zhang et al., 2012a, b). Nevertheless, other authors have reported changes in the viral capsids resulting in the failure of cell infection (Cozzi et al., 2023). Studying the antiviral activity of GTE, Falcó and colleagues found that the derivatives of EGCG, a flavonoid from GTE, are responsible for the antiviral effect of GTE at different pH, exerting subtle alterations of the capsid proteins while preserving the binding ability of human norovirus (Knight et al., 2013). Furthermore, improvements of the antiviral activity of GTE was enhanced by preparing the GTE solution 24 h before its use (aged-GTE). In addition, esters of EGCG with polyunsaturated fatty acids exhibited anti-hepatitis C virus activity (Yilmaz & Toledo, 2004). Bio-active extracts from immature persimmon fruits, containing significant phenolic contents (~ 11–27 mg gallic acid (GA)/g dry extract), displayed antiviral activity against MNV and HAV (Lipson et al., 2007). Similarly, extracts from *Posidonia oceanica* at concentrations of 0.5%, with polyphenols content of approximately 80 mg GA/g extract, were able to reduce the titers of both FCV and MNV by more than 2 log (Su et al., 2010a).

Medicinal and aromatic plants (MAPs) are also potential sources of natural bio-active phytochemical compounds, with polyphenols being the most relevant antioxidant molecules for food applications (Su et al., 2010b). Some of these MAPs have exhibited antiviral activity against MNV, FCV, and HAV. Duque-Soto and collaborators showed that polyphenols from *Ziziphora hispanica, Thymus longiflorus,* and *Origanum bastetanum* extracts reduced FCV titers by 4.2, 2.2, 2.2, and 2.4 log at 5 mg/mL, respectively, when tested at 25 °C. In the case of MNV, significant differences were observed for *Origanum bastetanum*, resulting in reductions by 1.5 log at 0.5 and 5 mg/mL (Duque-Soto et al., 2022). Similarly, Sandoval and collaborators evaluated the phenolic and antioxidant compounds extracted from arrayan (*Luma apiculata* (DC.) Burret) leaves against enteric viruses. MNV titers were reduced to undetectable levels, while HAV titers decreased by 2.4 log (Carrasco-Sandoval et al., 2022). The polyphenols extracted from the roots of Chinese liquorice (*Glycyrrhiza uralensis)*, such as glyasperin, glycyrin, 2′-methoxyisoliquiritigenin, licoflavonol, and glyasperin D, have been demonstrated to inactivate rotaviruses by directly inhibiting viral binding (Kwon et al., 2010).

About 50 Chinese plants were screened against human norovirus, and results showed that antiviral activity was determined by the inhibition of norovirus HBGA receptors bound by tannic acid (Zhang et al., 2012a, b).

#### Essential Oils and Compounds Thereof

EOs and their derivatives are aromatic compounds extracted from different plant parts. In the past, the industry has used EOs as flavoring agents and natural antimicrobials to improve food safety (ISO, 2019; Pinto et al., 2021).

Although there have not been many studies evaluating efficacy of EOs on human enteric viruses, published results suggest their use could be promising in the food sector. Carvacrol, lemongrass, allspice, mint oregano, or thymol are some of tested compounds (Table 1) (Carrasco-Sandoval et al., 2022; Chouhan et al., 2017; Duque-Soto et al., 2022; Elizaquível et al., 2013; Kwon et al., 2010; Zhang et al., 2012a, b). Significant reductions of more than 3 log were shown by thyme, clove or allspice Eos, inhibiting MNV and FCV replication (Carrasco-Sandoval et al., 2022; Duque-Soto et al., 2022; Elizaquível et al., 2013). Discrepant results have been reported on the efficacy of EOs against HAV. For example, thymol was tested by Sanchez and Aznar without success, however Battistini and collaborators, demonstrated the efficacy of lemon, grapefruit and rosemary cineole EOs on HAV, reducing viral titers by nearly 3 log (Battistini et al., 2019; Sanchez et al., 2015). A relevant factor to be considered when assessing the antiviral effect of EOs is the procedure used for the extraction, as different fractions and concentrations of active compounds may result. Additional variables to be considered are the seasonal variations due to the time of harvest, the plant germoplasms/variety, solubility and oxidation (Settanni et al., 2014).

The antiviral mechanism is specific of the type of EOs considered in general, the initial degradation of the capsid is followed by the subsequent damage of the viral RNA, finally preventing viral adsorption to host cells. Specifically, it has been reported that EOs denature structural glycoproteins and proteins of the viral particle, rendering them completely unable to infect (Chouhan et al., 2017).

#### Polysaccharides

Polysaccharides constitute a group of bio-active compounds with huge structural diversity, and some of them show antiviral activity. For example, *Stevia rebaudiana*, one of the most popular ingredients currently used by the food industry for its sweetening characteristics with low calorific value, has demonstrated antiviral activity against rotaviruses (Takahashi et al., 2001). Chitosan, derived from chitin, the second most abundant polysaccharide after cellulose, reduced FCV infectivity by more than 3 log (Akter et al., 2014; Amankwaah, 2013; Davis et al., 2012). In a recent study, the antiviral activity of aqueous fractions rich in β-glucans from *Pleurotus ostreatus* was evaluated against MNV to elucidate whether the extract composition and structural complexity of the β-glucans affected the antiviral activity. Overall, purified β-glucans significantly reduced MNV titers below the limit of detection, indicating that the greater structural heterogeneity of the polysaccharides had a positive effect on their antiviral properties. Consequently, the antiviral activity of the extracts was mainly attributed to the amount and type of polysaccharides, rather than the content of polyphenols and other low molecular weight compounds present in this type of natural compounds (Akter et al., 2014).

While not many natural compounds have been tested on HAV, three types of different carrageenans (iota-, lambda- and kappa carrageenan) were assayed in-vitro to assess their antiviral effect, resulting in up to 1.6 log reduction for ι-carrageenan at 500 mg/ml (Girond et al., 1991).

#### Organic Acids

The use of organic acids, specifically citric acid, is widespread in the food industry. Citrates, which are salts of citric acid used in food supplements and some medications, are also used. Studies on the effect of citrate on HAV particles revealed that epitopes became more accessible to antibodies, making them more susceptible to inactivation (Hansman et al., 2012). Additionally, Hansman and collaborators demostrated that citrate had the potential to inhibit the interaction of human norovirus with HBGAs, thereby blocking host infection. This supports the idea that foods rich in organic acids, such as citrus fruits, together with other bio-active compounds, may exert antiviral activity (Hansman et al., 2012; Li et al., 2013).

McLeod and colleagues investigated the application of citric acid and acetic acid to inactivate HEV on food and on food-contact surfaces. By measuring viral infectivity by cell culture, the authors concluded that while citric acid and acetic acid have potential applications to control HEV on food-contact surfaces, they are not suitable for direct use on food (McLeod et al., 2022).

Kowalczyk and coauthors performed assays where betulinic acid from hairy roots of *Senna obtusifolia* was tested against norovirus surrogates, showing 4 log reduction in FCV infectivity (Kowalczyk et al., 2021).

#### Proteins

In-vitro studies indicate that certain milk proteins interfere with viral infections. Lactoferrin, found in cow and breast milk, has been extensively researched for its ability to hinder viral infections, including MNV, poliovirus (PV) and rotavirus. This interference primarily occurs through lactoferrin binding to receptors on the host cell's surface or on viral particles. Furthermore, it disrupts viral protein structures and inhibits viral replication (Ishikawa et al., 2013; Kvistgaard et al., 2004; Pan et al., 2006).

## Natural Compounds in Food Applications

Within the food industry, various preservation methods, including heat treatment, salting, acidification, and drying, have been employed to extend the shelf life of food items and guarantee their safety by inhibiting the growth of specific microorganisms and inactivating human pathogens. Additionally, there is a growing interest in foods preserved with natural additives. These natural bio-active compounds can be directly incorporated into the product, applied to the food surfaces, integrated into packaging materials, or used in antiviral coatings, ensuring their efficacy in controlling viral contamination (Burt, 2004; Randazzo et al., 2018).

### Antiviral Activity of Natural Compounds in Food Applications

Currently, the exploration of natural compounds as potential antiviral agents in food applications represents a burgeoning area of research, with promising implications for food safety and public health. While this field is still in its early stages, a growing number of studies have begun to investigate the use of natural compounds to mitigate viral contamination in various food products. These investigations have yielded insights into the effectiveness of direct applications of different compounds in a range of food types, including jalapeno peppers, apple juice, milk, lettuce, and oysters. As a result, titer reductions for FCV, MNV, and HAV were reported when treated with GSE, carvacrol, and curcumin (Joshi et al., 2015; Sanchez et al., 2015; Su & D’Souza, 2013). Table 2 summarizes the main applications of natural antiviral compounds studied in recent years.

**Table 2:** Food applications using natural compounds with enteric antiviral activity

Food application	Natural compound	Concentration	Matrix	Virus	Method	Red	Refs.
Washing	Carvacrol	1%	Lettuce	FCV	TCID_50_	0.92	Sanchez et al. (2015)
				MNV		1.00	
	GSE	0.025%	Pepper	FCV	PFU	2.71	Joshi et al. (2015)
		0.1%		MNV		0.8	
	GTE	0.5%	Lettuce	HAV	TCID_50_	0.79	Randazzo et al. (2017)
		1%	Spinach	MNV		1.80	
Food-contact surfaces	GSE	0.2%	Stainless steel		PFU	0.56	Li et al. (2012)
	GTE	1%			TCID_50_	3.46	Randazzo et al. (2017)
			Glass			1.79	
				HAV		> 2.80	
	Acid citric	1%	Stainless steel	HEV	RT-qPCR	2.20	McLeod et al. (2022)
			Plastic			2.30	
		3%	Stainless steel			2.36	
			Plastic			2.32	
		5%	Stainless steel			2.44	
			Plastic			2.48	
Packaging	GSE	15%	Chitosan film	MNV	PFU	2.27	Amankwaah (2013)
	GTE	1:0.5 (Alginate:GTE)	Alginate		TCID_50_	2.25	Fabra et al. (2018)
		1:0.75 (Alginate:GTE)				2.79	
	CNMA	75 wt % (Zein/CNMA)	Polyhydrobutyrate	FCV		UDL	Fabra et al. (2016)
				MNV		2.75	
Coatings	GTE	1:0.7 (Alginate:GTE)	Strawberries			1.96	Fabra et al. (2018)
				HAV			
		1:0.7 (Carrageenans:GTE)	Raspberries	MNV		2.5	Falcó et al. (2019a, b, c)
			Blueberries			1.54	
	AITC	0.5% (Persian Gum:AITC)				2.04	Sharif et al. (2021)
	Larrea nitida	Agar				UDL	Moreno et al. (2020)
		Alginate					

*Red* reduction; *FCV* feline calicivirus; MNV: murine norovirus; *HAV* hepatitis A virus; *GSE* grape seed extract; *GTE* green tea extract; *CNMA* cinnamaldehyde; *AITC* allyl isothiocyanate; *UDL* under detection limits; *NTD* no titer decrease

### Natural Compounds as Sanitizers for Produce

**Fig. 1:**
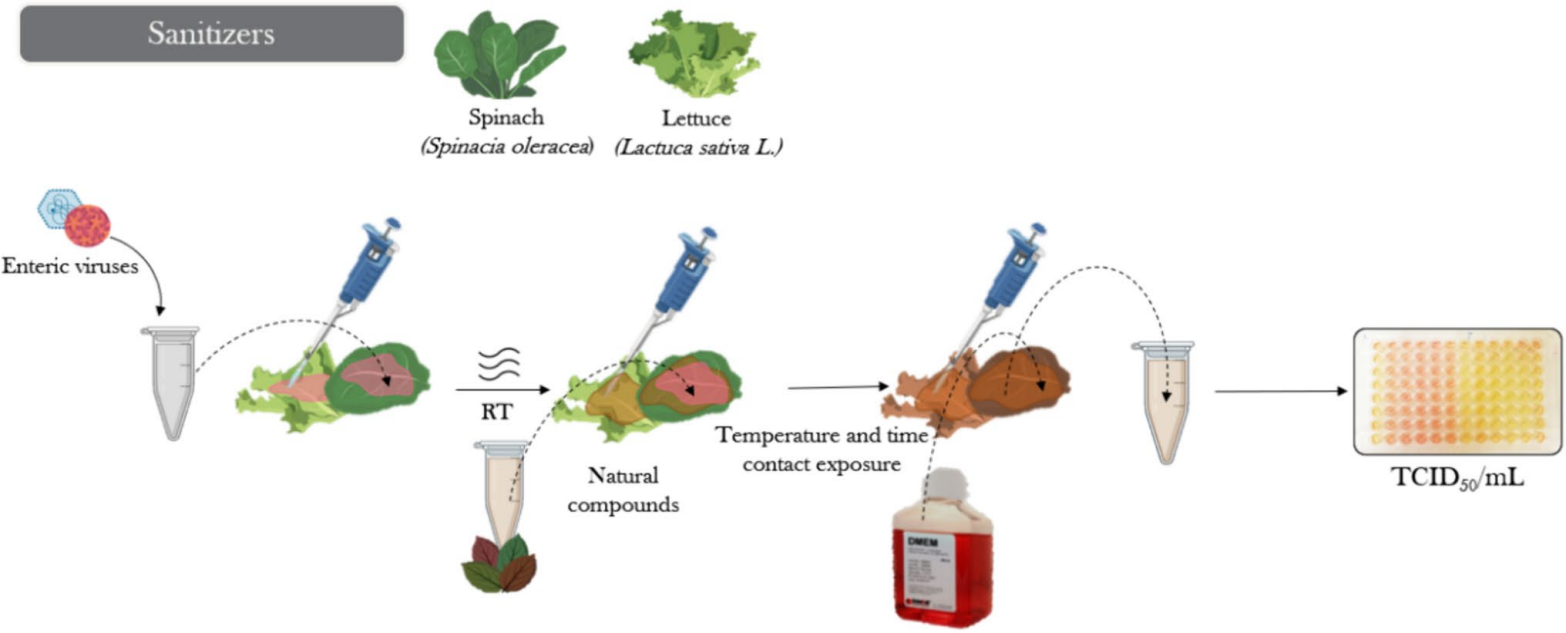
Overview of the experimental design for testing the antiviral activity of natural sanitizers

To date, chlorine has been the primary sanitizer used by produce industry. Current studies show that both chlorine and chlorine dioxide have a high capacity to inactivate human enteric viruses or their surrogates, such as HAV, human norovirus, MNV, TV and MS2 coliphage, when a minimum concentration of sanitizer and a given contact time are ensured (Allende et al., 2024; Dunkin et al., 2017; Falcó, et al., 2023a, b). Despite the efficacy shown by these compounds, peracetic acid, widely employed to control bacterial contamination, does not yield the same conclusion. The European Union and the USA are considering banning or reducing the use of chlorine and chlorine dioxide because of the chemical by-products generated (Van Haute et al., 2014). Thus, there is an increasing interest in the use of natural antiviral compounds in food processes, particularly in washing steps, to pursuit safer food processing methods and minimized risk for consumers. So far, carvacrol, GSE, and GTE are among the most popular natural compounds used as antiviral sanitizers (Fig. 1). In a study conducted by Sánchez et al. (2015), the antiviral activity of carvacrol was evaluated during the washing of lettuce, revealing reductions of nearly 2 log and reaching the limit of detection for norovirus surrogates after 30 min of exposure to a 1% carvacrol solution. Even more remarkable results were observed when GSE was used to disinfect peppers and lettuce, with viral titers for FCV, MNV, and HAV decreasing by 5.0, 1.2, and 1.2 log, respectively, after 5 min of treatment (Su & D’Souza, 2013). In addition, GTE was evaluated as a natural disinfectant for produce. A 10 mg/ml GTE solution reduced MNV and HAV titers in lettuce and spinach by more than 1.5 log after a 30-min treatment (Randazzo et al., 2017).

In spite of these promising applications, more studies are still needed to define treatment times and temperature conditions, adapting natural compounds to specific operational conditions.

### Natural Disinfectants for Decontaminating Food-Contact Surfaces

As commented previously, cross-contamination through food-contact surfaces is an important source of human enteric virus transmisión (Sanchez et al., 2015; Van Haute et al., 2014). Food surfaces are susceptible to contamination through direct contact with body secretions, lack of hygiene from food handlers, or aerosols generated by talking, sneezing, coughing, or vomiting. It is now well-established that chemical sanitizers exert strong antiviral activity on contaminated food-contact surfaces (Su & D’Souza, 2013; Van Haute et al., 2014). While their efficacy has been widely demonstrated, chemical sanitizers may require additional washing steps to ensure the removal of chemical residues that could potentially contaminate the food.

**Fig. 2:**
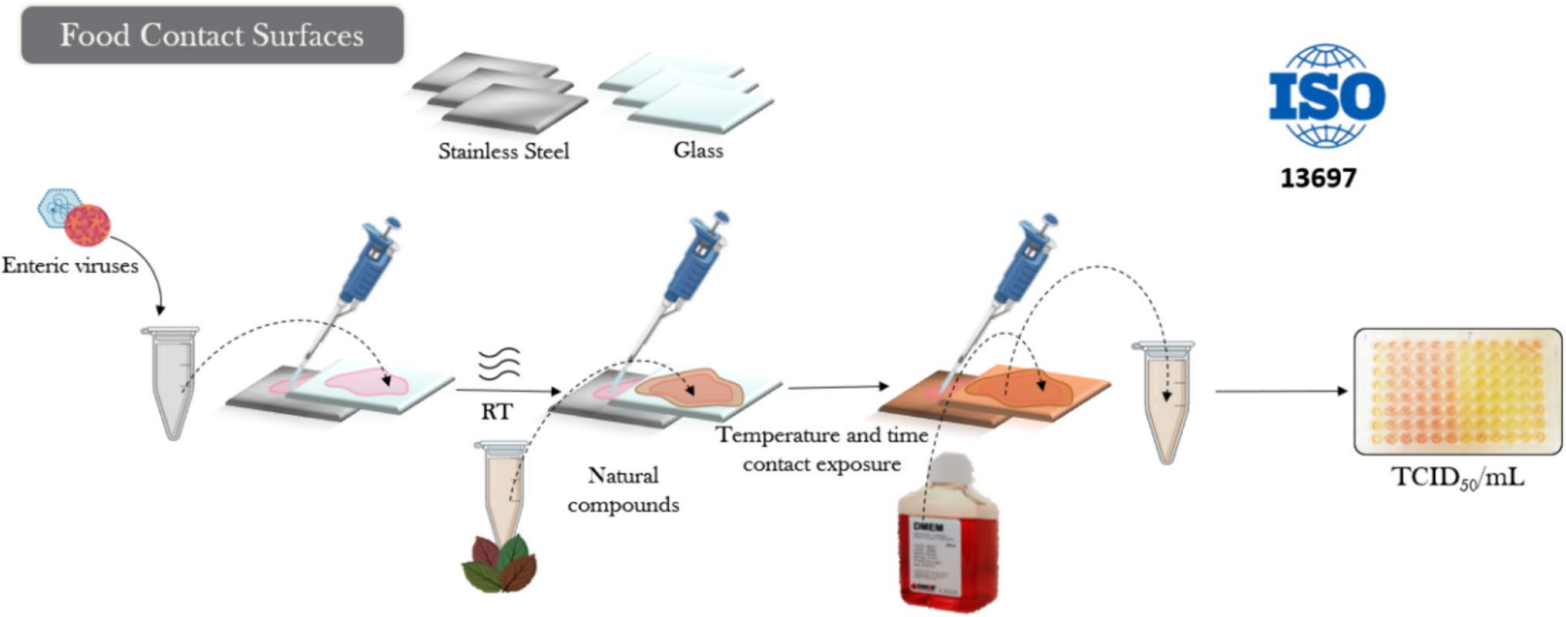
Overview of the experimental design for testing the antiviral activity of natural disinfectants for decontamination food-contact surfaces

Nevertheless, despite the importance of alternatives for cleaning surfaces, there are few studies assessing the use of natural compounds as sanitizers, except for GSE and GTE (Table 2, Fig. 2). GSE, applied at 1 mg/mL for 30 s, effectively reduced Aichi virus (AiV) to undetectable levels under clean conditions. In the presence of organic load, simulating unclean conditions, GSE concentrations of 2 and 4 mg/mL significantly reduced AiV infectivity (Abad et al., 2001). However, under conditions simulating real-world applications in the food industry, the efficacy of 2 mg/mL GSE on stainless steel surfaces contaminated with MNV was limited after 10 min (Joshi & D’Souza, 2021). Additionally, 10 mg/mL GTE for 30 min notably reduced MNV by 1.5 log and completely inactivated HAV on stainless steel and glass surfaces, complying with the ISO 13697:2001 standard (Randazzo et al., 2017). Citric and acetic acids were demonstrated to inactivate HEV on food-contact surfaces. Specifically, HEV-contaminated plastic and stainless steel surfaces were treated with acetic or citric acid at 1, 3, or 5%. Viral infectivity was reduced by more than 2 log as determined by cell culture, indicating that citric and acetic acids have potential applications to control HEV on food-contact surfaces (McLeod et al., 2022).

Overall, the above-mentioned studies revealed differences depending on the type of surface, highlighting the limited transferability of the results because of the high concentration and extended exposure/contact time, which may not be suitable for real scenarios.

### Bio-Active Packaging and Coatings

While the primary approach to preventing foodborne viral infections involves adhering to good hygienic, agricultural, and manufacturing practices active packaging stands out in the realm of food technology as an innovative solution, catering to consumer demands for fresh, ready-to-eat food while aligning with global market trends (Vojir et al., 2012). Numerous studies have established the effectiveness of active packaging in prolonging the shelf life of food items and controlling foodborne pathogenic bacteria.^111^ Nowadays, materials endowed with antimicrobial properties are widely utilized in the food industry, with several commercially available options (Seymour & Appleton, 2001).

Despite extensive research on the effectiveness of antimicrobial packaging against foodborne pathogenic bacteria and molds, investigations into their efficacy against human enteric viruses has only been reported in recent times. Thus, the development of materials enriched with antiviral natural compounds for food applications is emerging as an innovative approach that holds potential in preventing both cross and recontaminations. The resulting antiviral effect could be due to the material itself or the inclusion of antiviral compounds within the structure of the material that comes into contact with the contaminated food. In addition, the material could act as a carrier of the antiviral compounds, ideally gradually releasing them as volatile substances (Tiwari et al., 2009). Thus, packaging and coating materials can serve as an excellent vehicle for antiviral compounds in many fields within the food industry, such as food packaging, food-contact surfaces, and edible coatings (ISO, 2019).

#### Natural Antiviral Packaging

**Fig. 3:**
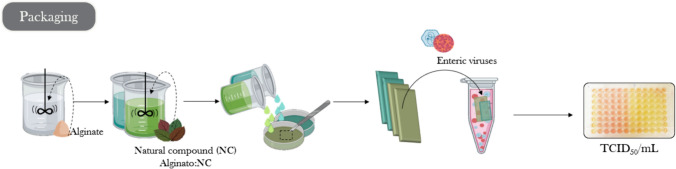
Overview of the experimental design for testing the activity of natural antiviral packaging

Edible films containing GTE or GSE have been formulated using different matrices and tested, as shown in Fig. 3 (Fabra et al., 2018; Falcó et al., 2019a, b, c). For instance, Fabra et al. incorporated cinnamaldehyde into a polyhydrobutyrate matrix, achieving significant reductions in MNV and FCV, although HAV proved more resistant (Fabra et al., 2016). By adapting the ISO 22196:2011 the authors estimated reductions of 2.75 log for MNV and a complete inactivation for FCV, while HAV proved to be resistant. Amankwaah (2013) explored the use of GSE in chitosan films, achieving substantial reductions in MNV titers, particularly with high GSE concentrations. Fabra et al. developed edible films with GTE and GSE in an alginate matrix, displaying promising antiviral activity (Fabra et al., 2018).

#### Natural Antiviral Coatings

**Fig. 4:**
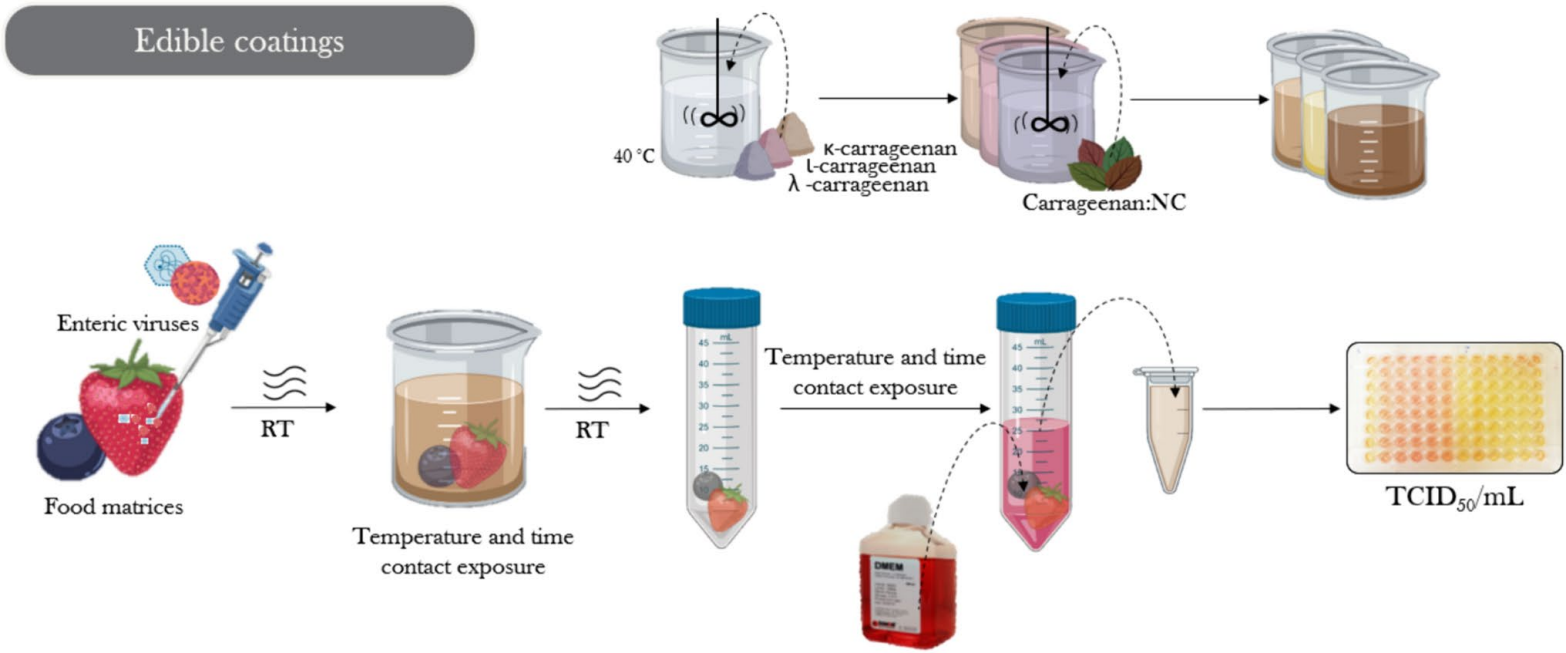
Overview of the experimental design for testing the activity of natural antiviral edible coatings

Edible coatings have emerged as a novel approach to control pathogens in raw foods like berries, which are susceptible to contamination by human enteric viruses (Fig. 4). Evaluating the efficacy of GTE-coated berries revealed the inactivation of MNV and HAV during storage at 10 and 25 °C, although this effect varied with the type of berry (Fabra et al., 2018). Strawberries have been used as models for testing antiviral coatings, including κ-, ι-, and λ-carrageenan with GTE, effectively inactivating MNV and HAV (Falcó et al., 2019a, b, c). Similarly, the study explored the antiviral properties of natural compounds in blueberry coatings, highlighting the enhanced efficacy of allyl isothiocyanate (AITC), especially at 37 °C, and the potential of agar and alginate-based coatings with or without the antiviral extract to inactivate enteric viruses, thereby enhancing food safety (Fabra et al., 2018; Falcó et al., 2019a, b, c; Sharif et al., 2021).

## Hurdle Technologies Involving Antiviral Natural Compounds

In the field of food technology research, the increasing demand for green food-processing technologies with improved sustainability (e.g., reduced energy/water consumption) and enhanced ability to prevent infections has led to the development of groundbreaking hurdle approaches, also known as hurdle technologies. These food processes combine a variety of technologies that constitute successive obstacles or barriers able to either eliminate the presence of pathogens or significantly reduce their presence. This approach has helped pave the way for new food safety standards. The combination of different hurdles results in pronounced inactivation efficacy due to additive or synergistic effects. In the hurdle effect, overall pathogen inactivation is not just the sum of the different preservative factors (additive effect), but it is even greater given the synergistic activity of the treatments (synergistic effect) (Gurtler et al., 2019; Leistner & Gorris, 1995).

Among many examples, the use of natural antimicrobials has been combined with mild processing techniques to minimize the severity of food processing while achieving the inactivation of foodborne pathogens (Del Nobile et al., 2012). This approach results in cost savings, maintenance of food safety, and preservation of nutritional and sensory attributes.

Traditionally, thermal treatments have been the most commonly applied technology for food preservation in both domestic and industrial settings (Gurtler et al., 2019). Unfortunately, high temperature treatments negatively impact food quality, decreasing both the nutritional and sensory value of foods (Leistner & Gorris, 1995; Peng et al., 2017). Thus, combining mild thermal treatments with additional hurdles, such as antimicrobial compounds, has been pointed out as a solution able to preserve food quality while ensuring food safety. This combination of technologies has been described as chemically-assisted low-temperature pasteurization or heat sensitization (Butot et al., 2008; Koskiniemi et al., 2013; Peng et al., 2017). The synergistic effect of natural compounds and food processing technologies has been reported for heat treatments coupled with curcumin, gingerol, GSE, or GTE on TV, MNV or HAV (Falcó et al., 2020; Patwardhan et al., 2020).

Patwardhan and colleagues investigated the effect of heat sensitization using curcumin, gingerol (from ginger), and GSE, on HAV and TV. Decreased *D*-values for TV and HAV were observed when heat treatments were applied in combination with each of the three natural compounds. Moreover, the linear model showed significant differences between the *D*-values of HAV and TV with and without the extracts for most tested temperatures. Thus, the authors concluded that these compounds can potentially lower temperature and time regimens needed to inactivate HAV and TV (Patwardhan et al., 2020).

In trials with the same purpose, Falcó and colleagues tested a preservation approach based on the use of aged-GTE and mild heat treatments to inactivate MNV and HAV in artificially contaminated fruit juices (a mixed fruit juice and an apple juice) (Falcó et al., 2020). Specifically, the authors combined mild heat treatments at 40, 50, or 63 °C with aged-GTE and reported that MNV titers were lower than those resulting from thermal treatment alone. These findings indicated a relevant synergistic antiviral effect of aged-GTE combined with mild heat treatments for MNV, which was not confirmed for HAV.

High hydrostatic pressure processing (HPP) is a nonthermal processing technique that has emerged as a promising technology to preserve a variety of food products, including fruit jams, orange juice, salsa, ready-to-eat meats, and oysters. In addition, inactivation studies have demonstrated the effectiveness of HPP to control viral pathogens, including HAV and human norovirus surrogates (Govaris & Pexara, 2021; Huang et al., 2020; Kingsley et al., 2007). The HPP technology was tested in combination with aged-GTE to inactivate HAV, MNV and human norovirus (Falcó et al., 2023a, b). Interestingly, the synergistic effect was demonstrated in buffered suspension against human norovirus GI by a binding assay and against norovirus GII by replication on HIE. Furthermore, HPP combined with aged-GTE was successfully tested to inactivate MNV and HAV in apple and horchata (a traditional beverage from Valencia, Spain) juices. Interestingly, the kinetic inactivation data reported by the authors corroborate the different sensitivity of the two viruses tested, with HAV being more sensitive than MNV (Falcó et al., 2023a, b, 2019a, b, c).

All these research findings strongly support the feasibility of adding natural compounds with antiviral activity to reduce the operating conditions (e.g., temperature, pression, time) of processing technologies, ultimately preserving food quality while guarantying food safety.

## Conclusions and Research Future Needs

Reviewed data indicates that a broad range of natural compounds exert antiviral activity in in-vitro tests. Experimental evidence on human norovirus and HEV inactivation by GRAS substances is still limited, even though studies exploiting novel replication models (e.g., HIE, zebra fish) are expected to be increasingly reported. However, research on food applications and the validation of their use under pilot or commercial conditions is very limited. In line with latest released FAO and WHO reports on hazards in produce, the general consideration is that there is still a need to test the antiviral activity of natural compounds in industrial applications and real-scenario settings.^132^ Furthermore, a more thorough examination of the impact of natural antiviral compounds on shelf life and sensory quality is needed, especially when their use is combined with additional preservation techniques.

## Data Availability

All data were obtained from publicly available information.
